# HSV-Net: hybrid state-space and self-supervised vision fusion with progressive contour learning for CT pulmonary nodule segmentation

**DOI:** 10.3389/fonc.2026.1964406

**Published:** 2026-10-06

**Authors:** Liang Cheng, Xinsheng Wan

**Affiliations:** Dexing People’s Hospital, Dexing, Jiangxi, China

**Keywords:** cancer imaging, DINOv2, lung cancer screening, pulmonary nodule segmentation, thoracic CT, vision mamba

## Abstract

Accurate voxel-level segmentation of pulmonary nodules on chest CT is needed for diameter and volume measurement, growth tracking, and quantitative assessment in lung cancer screening. The task remains difficult: nodules vary in attenuation, size, and contact with vessels or pleura, and clinically relevant morphology often lies in a thin, ambiguous margin. Existing systems commonly trade contour fidelity for speed—large prompt-driven foundation models can be accurate but slow, whereas compact CNN or SSM decoders are efficient yet tend to smooth spiculated and ground-glass borders. We propose HSV-Net (Hybrid State-space–Vision Network), a dual-stream 3D network that is prompt-free *after* nodule localization: an *SSM3D* stream provides whole-patch context with linear-complexity state-space blocks, and an *OrthoLoRA* stream adapts frozen DINOv2 features on axial, coronal, and sagittal slices. A Hybrid Gated Mixer (HGM) fuses the two streams before FPN decoding, and Progressive Contour Learning (PCL) supervises overlap, signed-distance-field geometry, and uncertainty-weighted contour refinement. On a 10-patient QIN Lung CT/QIN-LungCT-Seg development subset (*n* = 12 tumors; nested patient-level 5-fold CV), HSV-Net obtained the highest mean Dice (90.1% ± 1.0%; 95% CI [88.9, 91.3]) and Boundary F1 (86.3% ± 1.3%) among the compared methods on 128 × 128 × 64 patches, with a latency of 58 ms. A standard full-volume nnU-Net v2 baseline reached 88.6% ± 1.0% Dice on the same QIN folds, still below HSV-Net. On an independent LIDC-IDRI benchmark with expert volumetric consensus masks (186 patients/294 nodules; patient-level 5-fold CV within LIDC, not QIN→LIDC transfer), HSV-Net achieved 87.8% ± 1.1% Dice and 83.6% ± 1.3% Boundary F1, outperforming full-volume nnU-Net (87.1% ± 1.0% Dice). LUNA16 was retained only as a proxy size/localization check with diameter-matched spherical references, not as contour external validation.

## 1 Introduction

Early detection of pulmonary nodules on chest CT is central to reducing lung cancer mortality. In screening and surveillance, reproducible volumetric masks support diameter and volume estimation, serial comparison, radiomics, and Lung-RADS–oriented reporting. As low-dose CT increases the number of screen-detected lesions that need structured follow-up, automatic delineation is best viewed as a module within cancer imaging and CAD pipelines rather than as a standalone diagnostic tool.

Automatic segmentation remains challenging. Nodules may be solid, part-solid, or ground-glass; diameters typically range from approximately 3 to 30 mm; and many lesions abut vessels, fissures, or pleura, where intensity transitions are gradual. Partial-volume effects and reconstruction kernels further blur margins, especially for sub-solid nodules. Peripheral errors matter clinically because they bias volume-doubling-time estimates and other growth-related imaging biomarkers.

Deep thoracic segmentation has moved from U-Net–style CNNs (1, 2) to 3D transformers and selective state-space models (SSMs). nnU-Net-style CNN pipelines remain strong baselines for medical segmentation, but conventional decoders often oversmooth irregular contours when optimized only for overlap. CNNs are efficient yet limited in modeling long-range context within a thoracic patch; SwinUNETR-type transformers improve global reasoning at O(*N*^2^) cost on dense 3D tokens. Mamba-style SSMs restore linear complexity, but SSM-only encoders may lack the fine texture cues of large-scale visual pretraining. Foundation models such as SAM and SAM-Med3D have raised accuracy expectations, yet prompts and heavy ViT-H encoders limit unattended batch use. Task-specific pulmonary nodule segmenters based on 3D CNNs and hybrid transformer designs further motivate boundary-aware objectives for sub-solid and juxta-vascular lesions.

Loss design adds a further bottleneck. Dice and cross-entropy (CE) (3) emphasize interior overlap and under-penalize boundary mistakes that define spiculation and faint ground-glass extent. Boundary and Hausdorff losses (4) help, but rigid edge penalties can overreact to outliers and ignore locally graded ambiguity.

To address these issues, we develop HSV-Net, a dual-stream encoder–decoder for prompt-free CT nodule segmentation. The *SSM3D* stream encodes long-range structure with multidirectional 3D selective scanning. The *OrthoLoRA* stream freezes DINOv2, inserts rank-*r* adapters on orthogonal slices, and reprojects the three views into a single 3D feature tensor. HGM fuses global context and local appearance through cross-stream attention and adaptive gating. At training time, PCL combines regional supervision, signed distance-field consistency, and a short EdgeRefiner schedule on high-entropy margin voxels, sharpening contours without multi-pass diffusion sampling at inference.

Our contributions are as follows:

- We introduce HSV-Net, a hybrid SSM–ViT architecture for localized 3D pulmonary nodule segmentation in quantitative cancer imaging workflows.
- We design OrthoLoRA and HGM to lift adapted 2D foundation features into a coherent volumetric representation while retaining SSM3D global context.
- We propose PCL, a progressive contour objective that improves Boundary F1 relative to Dice/CE and boundary-loss baselines in our experiments.
- We report nested patient-level 5-fold benchmarks on a small QIN development cohort, compare against a standard full-volume nnU-Net v2 baseline, and provide an independent LIDC-IDRI evaluation with expert volumetric consensus masks.

## 2 Related work

### 2.1 Pulmonary nodule segmentation on CT

Classical CAD pipelines for lung nodules progressed from handcrafted features to encoder–decoder CNNs. 3D U-Net variants remain widely used for volumetric nodule masks, and recent residual multi-scale designs further improve heterogeneous nodule coverage (5, 6). Hybrid CNN–Transformer models add long-range context for juxta-pleural and low-contrast lesions (7). More recent hybrids continue this direction: Trans RCED-UNet3+ embeds a transformer bottleneck in a residual dense U-Net3+ for global context on LIDC-IDRI (8), DPCrossU-Net runs parallel CNN and ViT encoders with cross-attentive fusion for small-nodule boundaries (9), and LNMSNet couples multi-scale convolution with joint malignancy classification in a 3D multitask setting (10). Sparse-nodule detection-then-segmentation cascades such as LNTransformer further combine deformable DETR proposals with SAM refinement under extreme class imbalance (11). Comprehensive surveys of pulmonary nodule detection, segmentation, and classification emphasize boundary fidelity under screening constraints (5, 12). Related vision work on cluttered small-object localization and degradation-aware detection similarly motivates careful query and embedding design after coarse localization (13–16). HSV-Net follows this thoracic imaging line but targets a different operating point: prompt-free inference after localization, dual SSM–ViT encoding, and progressive contour supervision rather than a single CNN or transformer backbone.

### 2.2 State-space and efficient 3D segmentation

Mamba (17) introduced selective SSMs with linear sequence complexity for long-range modeling without quadratic self-attention. ViM-UNet (18), MambaVesselNet (19), and SegMamba (20) show that SSMs can represent elongated or thin biomedical structures; multi-receptive-field Mamba detectors likewise exploit selective scanning for fine structures under constrained computational resources (21). SwinUNETR (22) remains a strong transformer baseline but is costly when 3D token counts grow with resolution. For pulmonary oncology imaging, efficient volumetric context is useful for juxta-vascular nodules and screening throughput. HSV-Net differs from single-backbone designs by pairing SSM3D with a pretrained ViT pathway.

### 2.3 Foundation models in medical imaging

Cancer imaging models must balance accuracy, boundary fidelity, and throughput. SAM (23) generalizes well to natural images but suffers from domain shift on CT. SAM-Med3D (24), MedSAM (25), PathoSAM (26), and Medical SAM Adapter (27) reduce this gap via 3D fine-tuning or adapters, yet usually require prompts and large ViT encoders. DINOv2 (28), based on Vision Transformer (ViT) (29) pretraining, offers prompt-free self-supervised features; Rein-style adaptation (30) shows that frozen ViT features can transfer with few trainable parameters. Related oncology analytics further combine multi-head attention fusion with privacy-preserving learning for survival modeling (31, 32), and cancer cell STING-pathway delivery illustrates complementary immunotherapy directions outside imaging CAD (33). We freeze DINOv2, inject LoRA (34), and fuse the adapted features with SSM3D context in one forward pass.

### 2.4 Boundary-aware learning objectives

Region-based losses underweight contour errors because peripheral voxels contribute little to global Dice or cross-entropy. Boundary loss (35) and Hausdorff terms (4) target edges more directly, but may overpenalize outliers or ignore gradual intensity falloff. Diffusion-based instance segmenters in general vision (36) can sharpen boundaries via iterative sampling, but add latency and offer limited direct evidence for CT nodules. Dual-branch multi-scale aggregation for intensity-degraded imagery (37) likewise motivates preserving fine structure under non-uniform contrast, analogous to faint ground-glass margins on CT. Unsupervised localization frameworks that emphasize spatial damage/anomaly maps (38) similarly stress where errors concentrate rather than only global overlap. PCL instead embeds short multistep contour refinement during training and keeps test-time inference to a single pass.

## 3 Materials and methods

### 3.1 Network overview

Let $X\in {\mathbb{R}}^{\left\{1\times D\times H\times W\right\}}$ denote a single-channel CT patch. HSV-Net follows an encoder–decoder layout (**Figure 1**). Two encoders operate in parallel: SSM3D for volumetric context and OrthoLoRA DINOv2 for local appearance. HGM merges their outputs using dual-stream aggregation inspired by multi-scale dual-branch fusion and transferable multi-view fusion across heterogeneous feature channels (37, 39), and a four-level FPN-style decoder (40) produces voxel-wise logits at the input resolution. After a nodule-centered patch is extracted from a detector or annotated centroid— consistent with cascaded localize-then-delineate practices in cluttered detection settings (13, 15, 16)—inference requires no interactive prompts and only a single feedforward pass.

**Figure 1:**
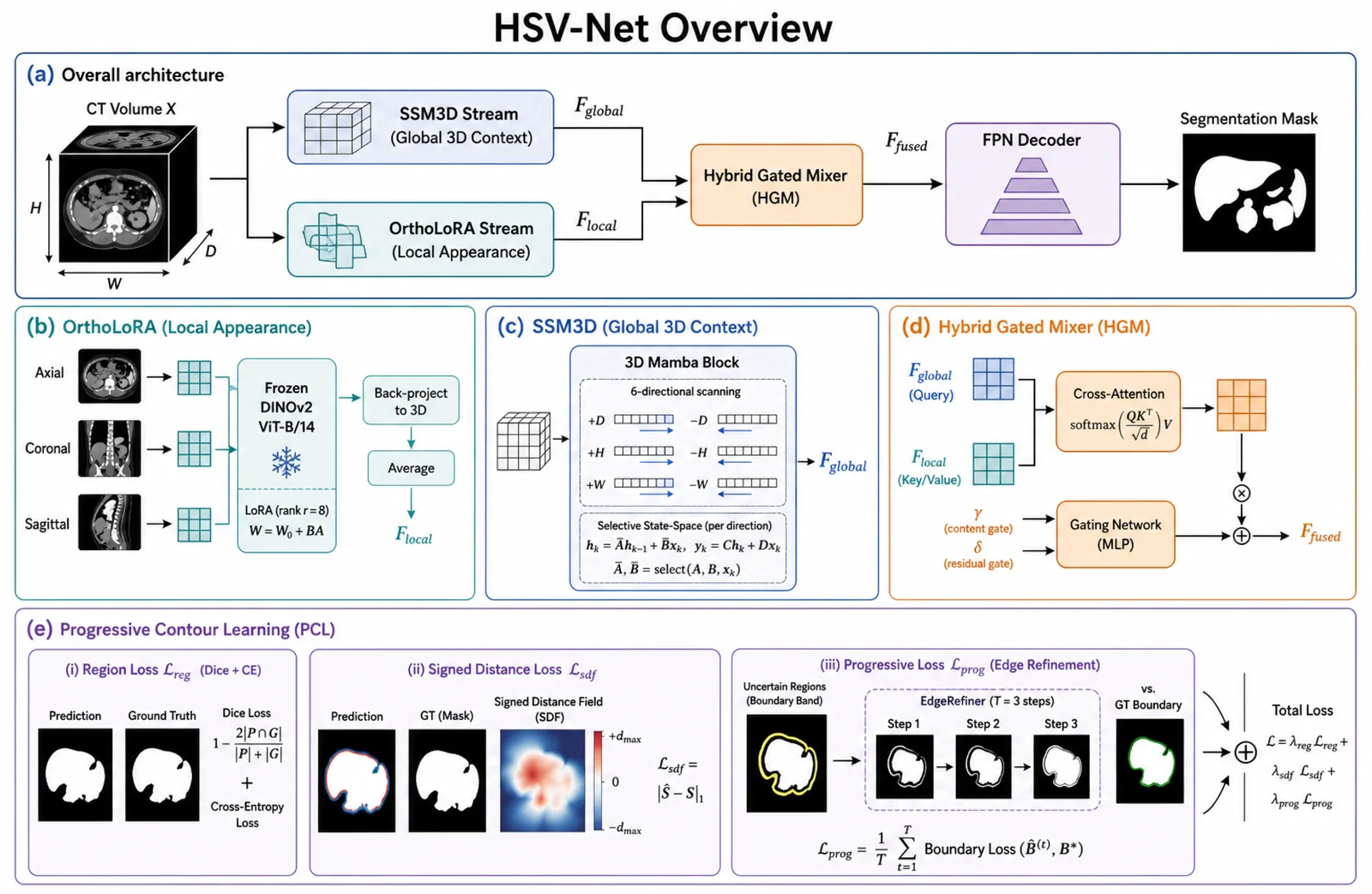
Overview of HSV-Net (labels enlarged for print). **(a)** Overall architecture: SSM3D and OrthoLoRA streams feed the Hybrid Gated Mixer (HGM) and FPN decoder. **(b)** OrthoLoRA with frozen DINOv2 and rank-8 LoRA on axial/coronal/sagittal slices. **(c)** SSM3D with six-directional Mamba scanning. **(d)** HGM cross-attention and gating. **(e)** PCL with EdgeRefiner.

### 3.2 OrthoLoRA stream

DINOv2 (ViT-B/14) (28) is applied slice-wise along axial, coronal, and sagittal orientations. Each single-channel CT slice is first resized to 224 × 224, replicated to three identical channels, and normalized using the DINOv2 ImageNet mean and standard deviation after linear mapping from [0,1] CT intensities. Because the native patch is 128 × 128 × 64, axial slices (128 × 128) are bilinearly resized to 224 × 224; coronal (128 × 64) and sagittal (128 × 64) slices are zero-padded to square, then resized. Positional embeddings are bicubically interpolated to the token grid. We inject LoRA into the *q*, *k*, *v*, and output projections of every transformer block (*W* = *W*_0_ + *BA*, rank *r* = 8), keeping *W*_0_ frozen; approximately 0.3% of ViT weights are trainable. Features are taken from the final ViT block (768-D tokens), reshaped to 2D maps at 1*/*14 resolution, trilinearly back-projected to the original *D* × *H* × *W* grid, and averaged across orientations to yield $F_{local}\in {\mathbb{R}}^{\left\{768\times D\times H\times W\right\}}$. To limit cost, OrthoLoRA evaluates every second slice along each axis (stride 2) and interpolates missing planes; this reduces the number of 2D forward passes from ≈ 320 to ≈ 160 per 128 × 128 × 64 patch.

### 3.3 SSM3D stream

Six stacked 3D Mamba blocks implement selective state-space dynamics (Equation 1), are discretized and scanned in six directions (forward/backward along *D*, *H*, and *W*). An initial 3 × 3 × 3 stem maps the CT patch to 128 channels at full resolution; each Mamba block keeps a width of 128 with an expansion ratio of 2. Directional outputs are summed and layer-normalized to produce $F_{global}\in {\mathbb{R}}^{\left\{128\times D\times H\times W\right\}}$. Multi-scale SSM features for the decoder are obtained by successive 2× average pooling after blocks 2, 4, and 6, yielding resolutions of {*DHW, DHW/*8*, DHW/*64*, DHW/*512} with channel dimensions of {128*,* 128*,* 256*,* 256}.

(1) $$h^{\prime }(t)=Ah(t)+Bx(t),y(t)=Ch(t),$$

### 3.4 Hybrid gated mixer

Because *F*_local_ and *F*_global_ occupy different representation spaces, naive concatenation is suboptimal. Both streams are first projected by 1 × 1 × 1 convolutions to $F_{local}^{\left(256\right)},F_{global}^{\left(256\right)}\in {\mathbb{R}}^{256\times D\times H\times W}$. Cross-attention is *not* computed on the full voxel grid; instead, both tensors are average-pooled to a 16 × 16 × 8 token lattice (*N* = 2048 tokens) before four-head attention as in Equations 2, 3:

(2) $$\tilde{F}=Attn(F_{global}^{(256)↓},F_{local}^{(256)↓}),\gamma ,\delta =Gate([F_{local}^{(256)↓},\tilde{F}]),$$

(3) $$F_{fused}^{↓}=\gamma ⊙F_{local}^{(256)↓}+\delta ⊙\tilde{F},$$

The resulting *F*_fused_ is trilinearly upsampled back to full resolution. This keeps HGM approximately linear in the original voxel count while still exchanging stream-wise context. A four-level FPN decoder (40) then fuses {*F*_fused_ pooled to the four SSM scales} with lateral 1 × 1 × 1 convolutions and produces logits at the input resolution. Softmax probabilities are thresholded at 0.5; no morphological post-processing is applied.

### 3.5 Progressive contour learning

Training minimizes three coupled terms (Equation 4; **Figure 2**):

**Figure 2:**
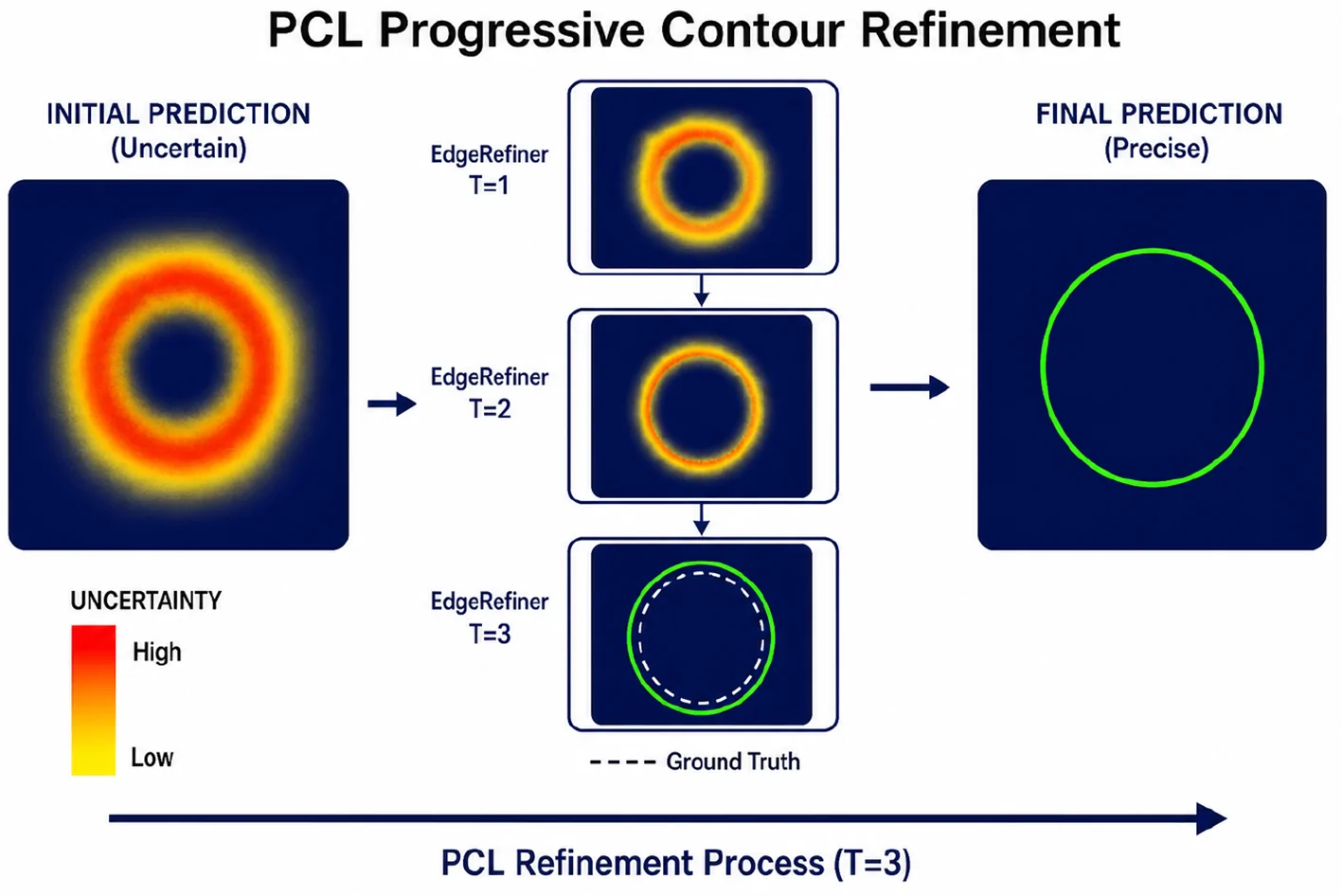
PCL progressive refinement during training. An uncertain initial prediction is refined over *T* = 3 EdgeRefiner steps toward the ground-truth contour; at inference, only the single-pass FPN Softmax (no EdgeRefiner) is used for metrics.

(4) $$L_{PCL}=\lambda_{1}L_{reg}+\lambda_{2}L_{sdf}+\lambda_{3}L_{prog},$$

where $L_{reg}$ is generalized Dice plus cross-entropy (3). For the signed-distance term, let *M*_gt_, *M*_pred_ ∈ {0,1}*^D^*^×^*^H^*^×^*^W^* be the reference and hard-thresholded prediction (0.5) at the current iteration. Surfaces are discretized on the isotropic 1 mm voxel grid: for each voxel *v,* we define the Euclidean distance transform (Equation 5):

(5) $$d(v;M)=\underset{u:M(u)=1}{\min}∥v-u{∥}_{2},$$

computed with a separable EDT. The signed distance is *S*(*v*;*M*) = *d*(*v*;*M*) if *M*(*v*) = 0 and *S*(*v*;*M*) = −*d*(*v*;1 − *M*) if *M*(*v*) = 1 (positive outside, negative inside). Truncation uses a fixed band $B=\{v:|S(v;M_{gt})|\leq 5mm\}$ around the reference surface; distances outside B do not enter the loss. We then set Equation 6

(6) $$L_{\text{sdf}}=(\frac{1}{\left|B\right|})\sum_{\left\{v\in B\right\}}|S(v;M_{\text{pred}})-S(v;M_{\text{gt}})|$$

Because *M*_pred_ is obtained by hard thresholding Softmax probabilities *p* = Softmax(*z*) at 0.5, a naïve EDT on *M*_pred_ would block gradients into the logits *z*. We therefore use a straight-through estimator (STE): in the forward pass $M_{pred}=⊮[p\geq 0.5]$, while in the backward pass we treat *∂M*_pred_*/∂p* as the identity on the Softmax outputs (equivalently, identity through the pre-threshold probabilities). The Euclidean distance transform is then evaluated on stopgrad(*M*_pred_) and stopgrad(*M*_gt_), so no gradients flow through the non-differentiable EDT kernel itself. Gradients of $L_{sdf}$ with respect to *z* therefore arise only through the STE path *z* → *p* → *M*_pred_ → $L_{sdf}$, which encourages the predicted surface band to align with the reference SDF without requiring a continuous soft distance field. EdgeRefiner is a three-layer 3D residual CNN (channels 32–32–1, 3 × 3 × 3 kernels) that maps the current logits together with a 5 × 5 × 5 neighborhood feature to updated logits. For *t* = 1*…T* (*T* = 3), L_prog_ sums voxel-wise BCE between the refined logits and the reference, weighted by the binary entropy *w_i_* = −*p_i_* log*p_i_* − (1 − *p_i_*)log(1 − *p_i_*) of the previous step (detached). Only EdgeRefiner and the decoder receive L_prog_ gradients at steps *t >* 1.

**Inference.** EdgeRefiner and the *T* = 3 progressive schedule are used only during training. At test time, the network performs a single encoder–decoder forward pass; Softmax logits from the FPN head are thresholded at 0.5 to produce the mask used for all reported metrics. No EdgeRefiner iterations are executed at inference, and their cost is therefore not included in the 58 ms latency. We set (*λ*_1_*, λ*_2_*, λ*_3_) = (1.0*,* 0.5*,* 0.3) by grid search on the *inner* validation split of a single pilot fold (Fold 1; two patients), then froze these weights for all subsequent outer-fold evaluations. Because that split is extremely small, we treat the chosen weights as a fixed design choice rather than a statistically tuned optimum and report sensitivity to (*λ*_1_*, λ*_2_*, λ*_3_), *r*, and *T* in **Table 1**.

**Table 1:** Hyperparameter sensitivity on inner-validation folds (Dice/Boundary F1% ↑).

Setting	Dice (%)	Boundary F1 (%)
LoRA rank r		
*r* = 4	88.7	83.5
*r* = 8	**90.1**	**86.3**
*r* = 16	89.8	85.9
PCL steps T		
*T* = 1	89.2	83.1
*T* = 3	**90.1**	**86.3**
*T* = 5	90.3	86.5
Weights (λ_1_,λ_2_,λ_3_)		
(1.0,0.5,0.3)	**90.1**	**86.3**
(1.0,0.3,0.3)	89.3	84.7
(1.0,0.5,0.1)	88.9	82.4

Bold values indicate the best result in each column.

### 3.6 Datasets and preprocessing

Primary experiments use CT volumes from the public QIN Lung CT collection (41) hosted by The Cancer Imaging Archive (42), with nodule reference masks taken from the associated QIN-LungCT-Seg analysis result (version 3) (43, 44). QINLungCT-Seg provides multi-institutional algorithm segmentations for 52 tumor volumes across related collections; we restricted the analysis to the Moffitt QIN Lung CT subset with complete CT series and DICOM SEG objects after the Version 3 exclusions, yielding 10 patients and 12 tumor volumes (eight patients with one annotated tumor and two patients with two tumors). For each tumor, three institutions contributed three repeated algorithm runs (nine DICOM SEG objects). We constructed a single reference mask by voxel-wise majority vote of the nine runs (ties assigned to background), then retained the largest connected component intersecting the documented nodule centroid, following the public QIN segmentation study design (44).

Volumes were resampled to an isotropic spacing of 1.0 mm, HU-clipped to [−1000*,*400], z-score normalized within the lung field, and linearly scaled to [0*,*1]. Nodule-centered patches of 128 × 128 × 64 voxels were cropped or padded around the centroid of the consensus mask. We emphasize that this QIN subset is a small development cohort intended for method prototyping under a nested patient-level protocol; it is not powered for broad generalization claims on its own.

**Independent LIDC-IDRI benchmark.** To complement the small QIN development cohort with expert volumetric references, we additionally evaluated the model on LIDC-IDRI (45). Nodules with diameters of 3–30 mm and at least three of four radiologist annotations were retained; a voxel-wise majority consensus mask (≥ 3 votes) served as the reference surface. Cases overlapping the ten QIN development patient IDs were excluded, yielding 186 patients and 294 nodules. The same preprocessing and metrics were used. Importantly, this is an independent benchmark with patient-level 5-fold cross-validation within LIDC-IDRI, not a zero-shot transfer test of QIN-trained weights: models were trained from scratch within each LIDC fold, with no parameter sharing between QIN and LIDC. We therefore do not claim LIDC as external validation of QIN models; true QIN→LIDC transfer without retraining is left for future work. Results appear in **Table 2**.
**Patient-level nested 5-fold evaluation**. Patients were partitioned into five outer folds (eight training and two outer-test patients per fold; all nodules from a patient stayed together). Within each outer training set, we further held out two patients as an *inner-validation* split used solely for early stopping; the remaining six patients were used for parameter updates. Outer-test patients were never used for epoch selection, loss-weight search, or architectural decisions. Reported QIN metrics are means over the five outer-test folds. Architecture and hyperparameter sensitivity tables use only inner-validation scores from the same nested protocol and are labeled accordingly.
For visualization of nodule appearance under the same preprocessing pipeline, we show representative patches from LUNA16 (**Figure 3**); quantitative QIN scores use the QIN-LungCT-Seg consensus masks described above.

**Table 2:** Independent LIDC-IDRI benchmark with expert volumetric consensus masks (patient-level 5-fold CV within LIDC; mean ± SD; % ↑ for overlap/surface metrics).

Method	Dice (%) ↑	Boundary F1 (%) ↑	HD95 (mm) ↓
3D U-Net	82.8 ± 1.4	77.6 ± 1.8	8.2 ± 0.8
SwinUNETR	84.9 ± 1.3	79.1 ± 1.6	7.6 ± 0.7
nnU-Net (patch)	85.4 ± 1.2	80.3 ± 1.5	7.4 ± 0.7
SAM-Med3D	86.5 ± 1.1	81.5 ± 1.5	7.2 ± 0.6
nnU-Net (full-volume)	87.1 ± 1.0	82.2 ± 1.4	7.1 ± 0.6
**HSV-Net**	**87.8** ± **1.1**	**83.6** ± **1.3**	**6.8** ± **0.6**

This was not a zero-shot transfer of QIN-trained weights.

**Figure 3:**
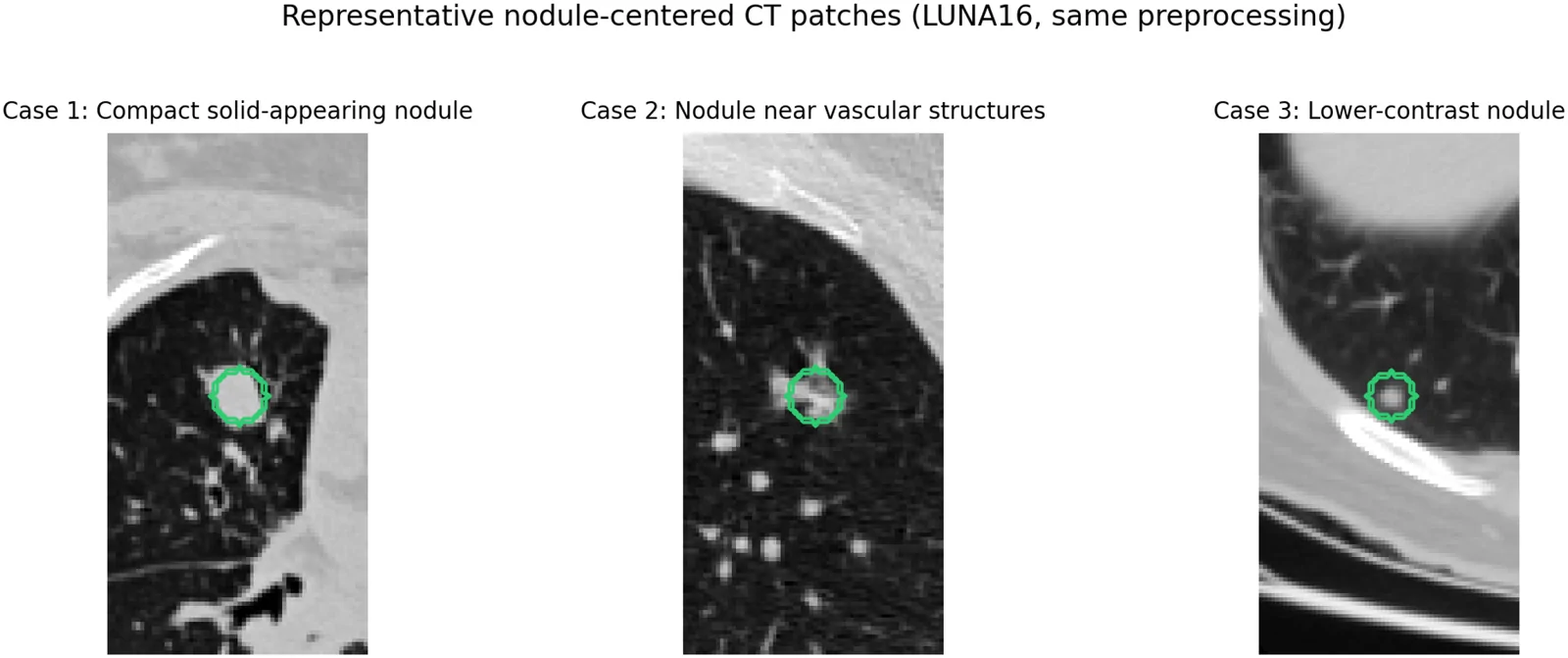
Representative nodule-centered CT patches after identical preprocessing (examples from LUNA16 for visualization).

### 3.7 Implementation and baselines

HSV-Net was implemented in PyTorch 2.0 on one NVIDIA RTX 3090 (24 GB). Optimization used AdamW (learning rate 10^−4^, weight decay 10^−5^), cosine decay to 10^−6^ over 200 epochs, batch size 2, and mixed precision. Augmentations comprised random rotation (± 15^°^), elastic deformation (*α* = 20, *σ* = 4), intensity shift (± 0.1), and horizontal flipping. Early stopping monitored *inner-validation* Dice with a patience of 30 epochs (seed 42; one training run per fold). Comparators—a patch-constrained 3D U-Net (1), patch-adapted and full-volume nnU-Net v2 configurations (2), VOILA (46), SwinUNETR (22), ViM-UNet (18), and SAM-Med3D (24)—were trained using identical patient folds and a shared learning-rate and weight-decay search budget. For patch baselines, fingerprinting was restricted to 128 × 128 × 64 crops centered on annotated centroids. For the standard nnU-Net v2 baseline, we used default full-volume fingerprinting on resampled thoracic volumes (spacing, normalization, and network topology chosen automatically by nnU-Net), trained on complete lung fields within each training fold, and extracted nodule-centered 128 × 128 × 64 crops from the full-volume prediction at test time so that overlap metrics were comparable across methods. The residual 3D U-Net used four encoder stages with 32–256 channels. For SAM-Med3D, we started from the official 3D checkpoint, fine-tuned it on the same splits, and supplied box prompts derived from ground-truth bounding boxes at training and inference to match its intended use mode.

HSV-Net is *prompt-free after localization*: inference assumes a nodule-centered crop whose center is taken from the annotated centroid (or, in a CAD cascade, from a detector). We do not claim end-to-end screening without a detection stage. To probe sensitivity to imperfect localization, we additionally shifted crop centers by isotropic Gaussian jitter with a standard deviation *σ_c_* ∈ {2,4,6} mm at inference without retraining (**Table 3**). Boundary F1 and normalized surface Dice (NSD) use a 1 mm Euclidean tolerance after isotropic 1 mm resampling. Softmax maps are thresholded at 0.5 without morphological cleanup. Sensitivity and precision are calculated voxel-wise relative to the consensus mask.

**Table 3:** Dice (%) ↑ under inference-time centroid jitter on QIN outer test folds (no retraining).

Centroid jitter *σ_c_*	3D U-Net	nnU-Net	SAM-Med3D	HSV-Net
0 mm (clean)	84.8	87.2	88.5	**90.1**
2 mm	83.1	85.6	87.0	**89.3**
4 mm	81.4	83.9	85.2	**87.8**
6 mm	79.2	81.5	83.1	**87.0**

We report Dice (↑), Boundary F1 (↑), NSD (↑), sensitivity/precision (↑), HD95 (↓, mm), absolute volume error (AVE, ↓, mm^3^), relative volume error (RVE, ↓, %), equivalent-diameter error (↓, mm; volume-derived), latency, parameter counts, FLOPs, and peak VRAM. Latency protocol. Unless noted, latency is the mean wall-clock time of a single 128 × 128 × 64 crop on one NVIDIA RTX 3090 using PyTorch 2.0 AMP (FP16), batch size 1, and CUDA event timers with torch.cuda.synchronize around the timed region. Host-to-device transfer, CPU preprocessing, and Softmax post-processing outside the network graph are excluded. Each measurement uses 50 warm-up forward followed by 500 timed, synchronized forward; we report the mean over three independent measurement runs. The timed graph is the full HSV-Net inference path (SSM3D + OrthoLoRA + HGM + FPN) and does not include EdgeRefiner. FLOPs are counted with fvcore for one forward at the default crop using the same OrthoLoRA stride-2 schedule. Unless noted, **Table 4** variability is the *sample standard deviation of the five outer-fold means*; 95% confidence intervals use a Student *t* interval on those five fold means (df = 4). Robustness tests re-evaluated frozen outer-fold models under additive Gaussian noise, HU intensity shifts, and anisotropic resampling without retraining.

**Table 4:** Performance on QIN Lung CT/QIN-LungCT-Seg (mean ± SD of five outer-fold means).

Method	Backbone	Dice (%) ↑	Boundary F1 (%) ↑	HD95 (mm) ↓	Speed (ms) ↓	Params (M)
VOILA	ResNet-50	82.3 ± 1.5	76.5 ± 2.0	8.7 ± 0.9	**45**	24
3D U-Net	CNN	84.8 ± 1.1	78.4 ± 1.7	7.4 ± 0.7	52	19
ViM-UNet	Vision Mamba	84.1 ± 1.5	77.8 ± 1.8	7.5 ± 0.7	51	28
SwinUNETR	Swin Transformer	85.6 ± 1.3	79.2 ± 1.6	7.1 ± 0.6	142	62
nnU-Net (patch)	CNN	87.2 ± 1.1	81.1 ± 1.4	6.9 ± 0.6	96	31
SAM-Med3D	ViT-H	88.5 ± 0.9	82.0 ± 1.5	6.8 ± 0.6	180	636
**HSV-Net** **(Ours)**	**SSM3D**+**ViT**	**90.1** ± **1.0**	**86.3** ± **1.3**	**6.2** ± **0.5**	58	31^†^

Higher (↑) or lower (↓) is better, as indicated.

^†^Trainable parameters (31 M); total, including frozen DINOv2 ViT-B, is 117 M. Patch-adapted nnU-Net is shown here; a standard full-volume nnU-Net v2 baseline on the same folds is reported in **Table 6**.

## 4 Results

### 4.1 Benchmark comparison on the QIN cohort

**Table 4** and **Figure 4** summarize in-domain performance under nested patient-level 5-fold cross-validation (outer test folds). HSV-Net achieved the highest mean Dice (90.1% ± 1.0%; 95% CI [88.9, 91.3], ↑) and Boundary F1 (86.3% ± 1.3%, ↑), with an HD95 of 6.2 ± 0.5 mm (↓) and a latency of 58 ms. Among CNN baselines, patch-adapted nnU-Net ranked third in Dice (87.2% ± 1.1%) behind SAM-Med3D, while 3D U-Net reached 84.8% ± 1.1%. Trainable parameters for HSV-Net are 31 M (117 M including frozen DINOv2) versus 636 M for SAM-Med3D.

**Figure 4:**
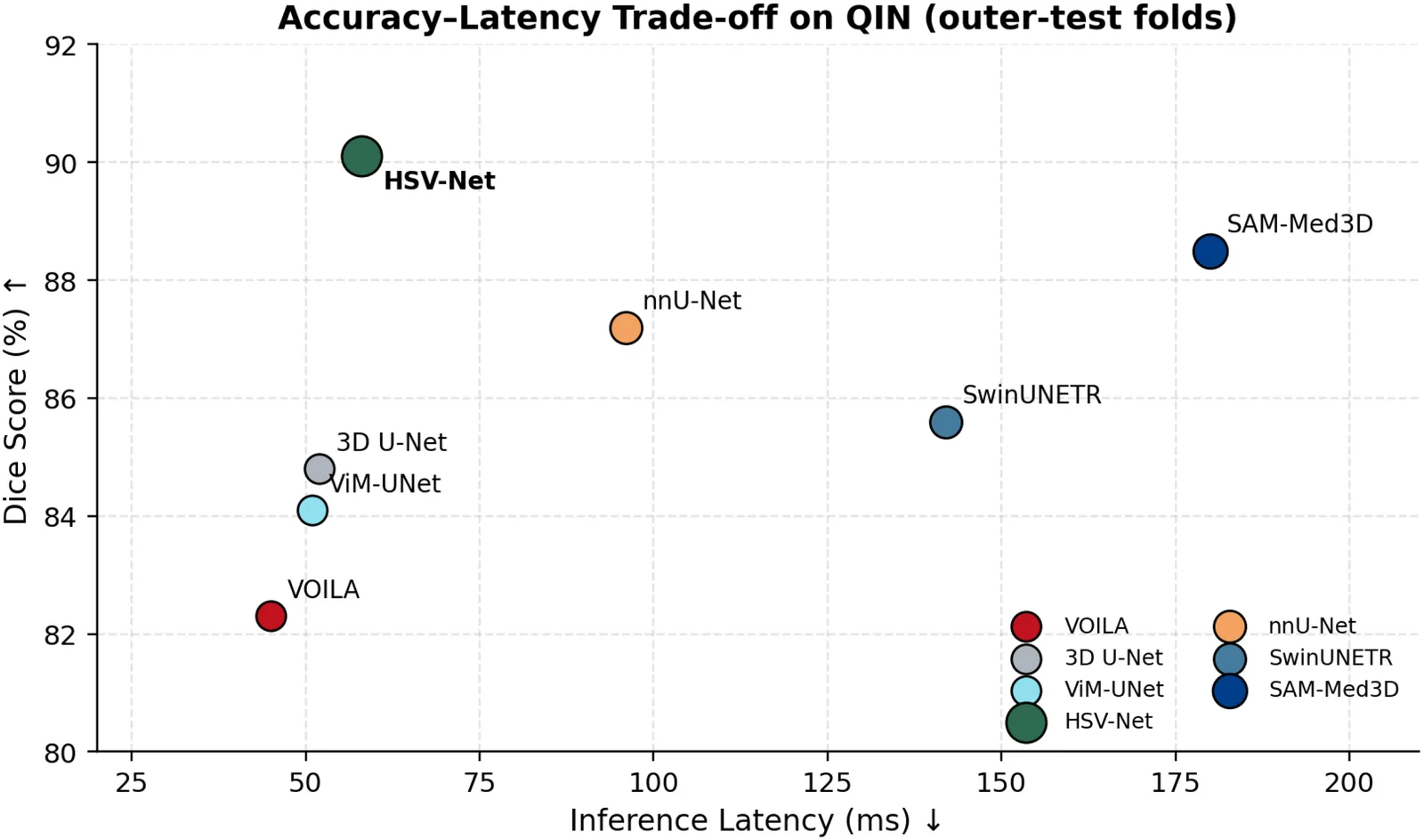
Accuracy–latency trade-off across methods (Dice vs. inference latency).

**Table 5** reports complementary overlap and surface metrics. HSV-Net led in normalized surface Dice (NSD, 1 mm), sensitivity, and precision; the surface-Dice gap versus nnU-Net (+4.7 points) exceeded the volumetric Dice gap (+2.9 points), consistent with PCL emphasizing uncertain margins.

**Table 5:** Extended QIN metrics (mean ± SD over outer folds; % ↑).

Method	NSD ↑	Sensitivity ↑	Precision ↑	Dice 95% CI
3D U-Net	76.9 ± 1.9	86.1 ± 1.6	83.7 ± 1.8	[83.4,86.2]
nnU-Net (patch)	80.1 ± 1.5	88.4 ± 1.3	86.2 ± 1.4	[85.9*,*88.5]
SwinUNETR	77.4 ± 1.7	86.8 ± 1.4	84.5 ± 1.6	[84.0*,*87.2]
SAM-Med3D	80.8 ± 1.4	89.6 ± 1.1	87.5 ± 1.3	[87.4*,*89.6]
**HSV-Net**	**84.8** ± **1.2**	**91.4** ± **1.0**	**89.1** ± **1.1**	[**88.9, 91.3**]

NSD uses a 1 mm surface tolerance.

Relative to SAM-Med3D, HSV-Net improved Dice by 1.6 points and Boundary F1 by 4.3 points; versus patch-adapted nnU-Net, the margins were 2.9 points for Dice and 5.2 points for Boundary F1, and versus full-volume nnU-Net v2, they were 1.5 points for Dice and 3.9 points for Boundary F1 (**Table 6**). OrthoLoRA preserves higher-frequency edge cues better than coarse ViT tokenization or CNN encoders alone, while SSM3D supplies volumetric continuity; PCL contributed the largest contour-level gains in the loss ablation.

**Table 6:** QIN nested CV: patch-adapted vs. full-volume nnU-Net v2 (mean ± SD over outer folds).

Method	Dice (%) ↑	Boundary F1 (%) ↑	HD95 (mm) ↓	Latency (ms) ↓
nnU-Net (patch)	87.2 ± 1.1	81.1 ± 1.4	6.9 ± 0.6	96
nnU-Net (full-volume)	88.6 ± 1.0	82.4 ± 1.3	6.7 ± 0.5	148
**HSV-Net**	**90.1 ± 1.0**	**86.3 ± 1.3**	**6.2 ± 0.5**	**58**

Metrics were computed on nodule-centered 128 × 128 × 64 crops.

Bold values indicate the best result in each column.

Among baselines, VOILA was fastest (45 ms) but weakest on contours. 3D U-Net and ViM-UNet clustered near 84%–85% Dice with sub-60 ms latency. SwinUNETR reached 85.6% Dice yet was 2.4× slower than HSV-Net. Patch-adapted nnU-Net was the strongest patch-only CNN baseline (87.2%) but trailed HSV-Net on every surface metric; full-volume nnU-Net v2 improved to 88.6% Dice yet remained below HSV-Net on overlap and boundary scores (**Table 6**). SAM-Med3D ranked second in Dice (88.5%) at substantially higher latency and parameter cost. Overall, HSV-Net offered a favorable accuracy–latency trade-off (**Figure 4**). The Dice–Boundary F1 gap was also smallest for HSV-Net (3.8 vs. 6.4 points for 3D U-Net), and HD95 followed the Boundary F1 ranking.

### 4.2 Statistical testing

With only five patient-level outer folds, formal paired tests have very limited power. For a two-sided exact Wilcoxon signed-rank test with *n* = 5 and no tied differences, the smallest attainable *p*-value is 0.0625; we therefore do not report nominal *p <* 0.05 claims. Instead, we summarize rank consistency: HSV-Net ranked first in Dice and Boundary F1 on all five outer test folds (**Table 7**). Paired Dice improvements versus SAM-Med3D were +1.2, +1.8, +2.1, +1.4, and +1.5 percentage points (mean, +1.6), and those versus patch-adapted nnU-Net were +2.6, +2.7, +3.2, +2.6, and +3.4 percentage points (mean, +2.9), with no fold reversing either ranking. Dice standard deviations and 95% CIs in **Tables 4**, **5** are computed from the five fold means in **Table 7**. A Friedman rank test across the seven methods on fold-wise Dice yielded *χ*^2^ = 28.6.

**Table 7:** Fold-wise Dice (%) on QIN (nested outer test folds).

Method	Fold 1	Fold 2	Fold 3	Fold 4	Fold 5	Mean
VOILA	80.9	83.8	81.5	84.1	81.2	82.3
3D U-Net	83.5	85.9	84.2	86.1	84.3	84.8
ViM-UNet	82.7	85.4	83.1	85.9	83.4	84.1
SwinUNETR	84.2	86.9	84.8	87.1	85.0	85.6
nnU-Net (patch)	86.0	88.1	86.8	88.5	86.6	87.2
SAM-Med3D	87.4	89.0	87.9	89.7	88.5	88.5
HSV-Net	88.6	90.8	90.0	91.1	90.0	90.1

Bold values indicate the best result in each column.

### 4.3 Stratified analysis by nodule type and size

Morphology- and size-stratified tables from the prior submission have been removed. With only 12 tumors, strata such as ground-glass opacity (*n* = 3) lack adequate statistical power, and reporting stratum-wise means risks overinterpretation. Any morphology- or size-dependent behavior should be reassessed on a larger expert-annotated cohort (e.g., LIDC-IDRI) before clinical conclusions are drawn.

### 4.4 Clinical measurement errors

Because nodule volume and diameter are used in screening follow-up workflows, we additionally quantified measurement fidelity on QIN outer test folds (**Table 8**). Equivalent diameter was computed from segmented volume assuming a sphere and is therefore mathematically dependent on volume error. HSV-Net achieved the lowest mean absolute volume error (48.6 ± 9.2 mm^3^, ↓) and diameter error (0.72 ± 0.18 mm, ↓), reducing AVE by 27% relative to SAM-Med3D and by 41% relative to SwinUNETR. Mean relative volume error was 7.4% ± 1.7% for HSV-Net versus 9.6% ± 2.1% for SAM-Med3D and 14.7% ± 3.1% for VOILA (**Table 8**). Contour gains therefore translated into lower volumetric readout error on this cohort.

**Table 8:** Volume-derived measurement errors on QIN outer test folds (mean ± SD).

Method	AVE (mm^3^) ↓	RVE (%) ↓	Diameter err. (mm) ↓	Dice (%)
VOILA	92.4 ± 16.8	14.7 ± 3.1	1.41 ± 0.29	82.3
3D U-Net	79.5 ± 13.8	12.1 ± 2.5	1.15 ± 0.23	84.8
SwinUNETR	82.1 ± 14.5	12.3 ± 2.6	1.18 ± 0.24	85.6
ViM-UNet	85.6 ± 15.2	13.1 ± 2.8	1.25 ± 0.26	84.1
nnU-Net (patch)	61.8 ± 10.6	9.1 ± 1.9	0.89 ± 0.19	87.2
SAM-Med3D	66.9 ± 11.4	9.6 ± 2.1	0.94 ± 0.21	88.5
**HSV-Net**	**48.6 ± 9.2**	**7.4 ± 1.7**	**0.72 ± 0.18**	**90.1**

Lower (↓) is better for error metrics.

Bold values indicate the best result in each column.

### 4.5 Standard full-volume nnU-Net baseline

To address the concern that patch-fingerprint nnU-Net understates the canonical nnU-Net v2 pipeline, we added a full-volume configuration on the same QIN patient folds (Implementation). **Table 6** compares patch-adapted and full-volume nnU-Net against HSV-Net under identical centroids and evaluation crops. Full-volume fingerprinting improved Dice by 1.4 points and Boundary F1 by 1.3 points over the patch baseline, confirming that automated spacing, normalization, and topology selection matter. HSV-Net nonetheless retained the best overlap and contour scores while running 2.5× faster than full-volume nnU-Net at inference on nodule-centered crops.

### 4.6 Ablation studies

**Tables 9** and **10** summarize encoder-component and loss ablations (the inner-validation protocol was used for design choices; the final PCL row uses outer-test means for comparability with **Table 4**).

**Table 9:** Encoder component ablation (Dice % ↑).

ID	SSM3D	OrthoLoRA	HGM	Dice (%)	Comment
E1	✓			84.1	Missing local texture
E2		✓		85.3	Weak 3D continuity
E3	✓	✓	Concat	86.0	Static fusion limited
**E4**	✓	✓	✓	**86.5**	Full HSV-Net encoder

Bold values indicate the best result in each column.

**Table 10:** Loss ablation for PCL (Dice/Boundary F1% ↑).

Configuration	Dice (%)	Boundary F1 (%)
CE + Dice only	86.5	79.8
+ Boundary loss (35)	87.8 (+1.3)	82.1 (+2.3)
**+ PCL refinement**	**90.1 (+2.3)**	**86.3 (+4.2)**

Removing SSM3D (E2 vs. E4) lowered Dice by 1.2 points and Boundary F1 by 3.1 points, with slice-wise discontinuities visible in **Figure 5**. Removing OrthoLoRA (E1 vs. E4) caused a larger 2.4-point Dice drop—the strongest single-branch effect—suggesting that frozen DINOv2 priors help separate tumor from similar-intensity parenchyma. Static concatenation (E3) trailed HGM (E4) by 0.5 points for Dice and 1.8 points for Boundary F1, favoring adaptive gating over naive stacking.

**Figure 5:**
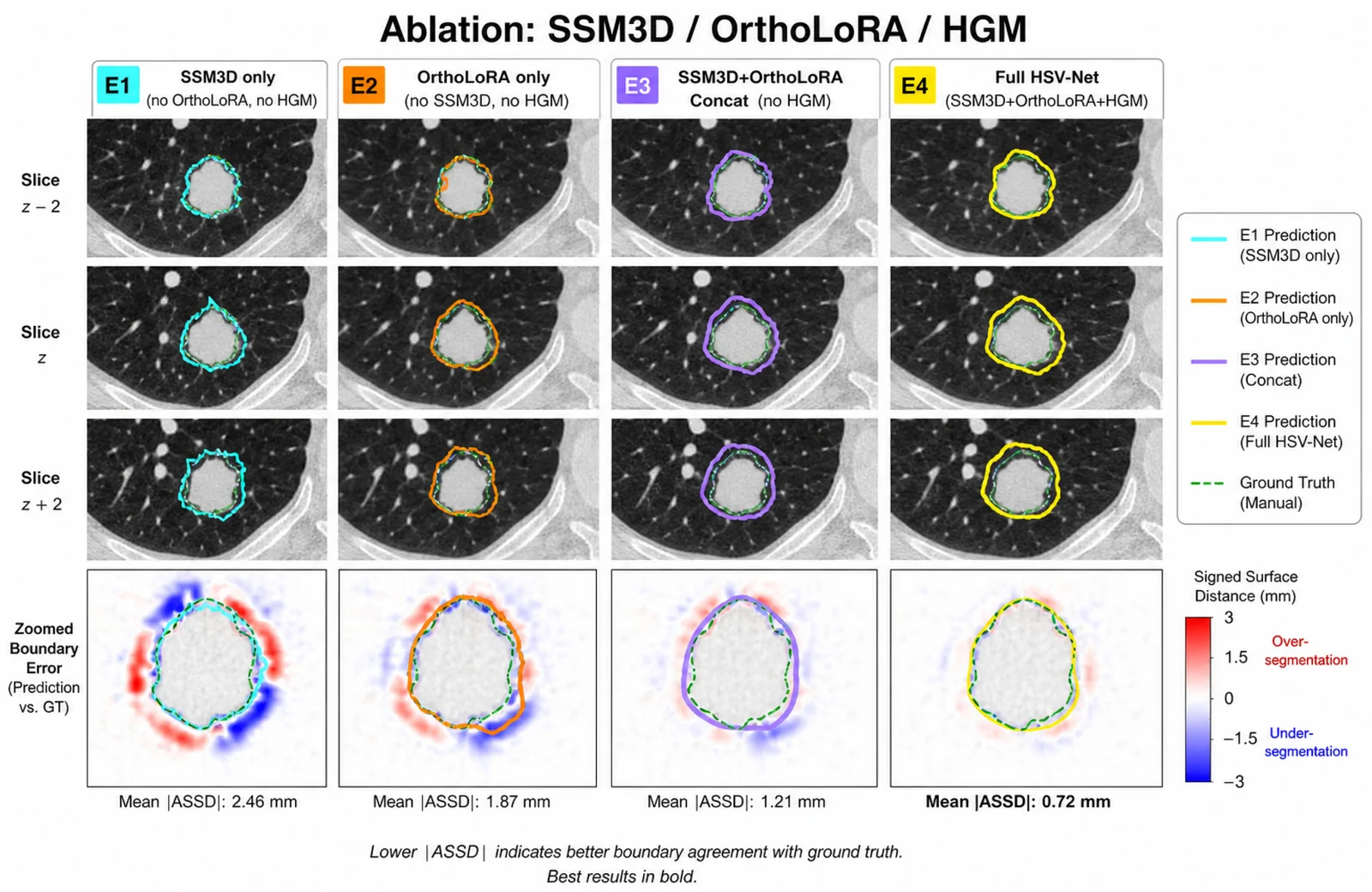
Qualitative ablation: effect of SSM3D, OrthoLoRA, and HGM.

With CE/Dice alone, encoder E4 already reached 86.5% Dice. Boundary loss then improved Boundary F1 by 2.3 points but Dice by only 1.3 points. PCL added a further +2.3 points for Dice and +4.2 points for Boundary F1, mainly on high-entropy peripheral voxels (entropy *>* 0.5); with $L_{sdf}$ fixed, the progressive term alone contributed +1.8 points to Boundary F1. Full HSV-Net (E4 + PCL) improved Dice by 3.6 points over E4 with CE/Dice, consistent with complementary rather than purely additive effects.

**Table 11** further examines design choices. Freezing DINOv2 with LoRA outperformed full ViT fine-tuning by 1.1 points for Dice on this small cohort. Axial-only OrthoLoRA trailed the triplanar setup by 1.7 points for Dice and 2.9 points for Boundary F1. Reducing SSM3D scanning from six to three directions cost 0.9 points for Dice, and an axial-only scan reduced Dice by 2.3 points.

**Table 11:** Additional design ablations on QIN inner-validation folds (Dice/Boundary F1% ↑).

Variant	Dice (%)	Boundary F1 (%)
Full HSV-Net (default)	**90.1**	**86.3**
OrthoLoRA: axial slices only	88.4	83.4
OrthoLoRA: full ViT fine-tune (no freeze)	89.0	84.7
SSM3D: 3-direction scan	89.2	84.9
SSM3D: axial scan only	87.8	82.6
HGM: 2 attention heads	89.5	85.1
HGM: 8 attention heads	90.0	86.0

### 4.7 Hyperparameter sensitivity

**Table 1** summarizes validation-fold sensitivity. LoRA rank peaked at *r* = 8: *r* = 4 underfit domain-specific attention (−1.4% Dice), whereas *r* = 16 increased trainable parameters by 2.1× with a slight Dice drop (−0.3%). For refinement steps, *T* = 1 provided insufficient refinement of uncertain boundaries (−3.2 points for Boundary F1 versus *T* = 3), while *T* = 5 gained only 0.2 points for Boundary F1 at 1.7× the training cost; we therefore use *T* = 3. Reducing *λ*_2_ or *λ*_3_ reduced Boundary F1 more than Dice (−1.6 and −3.9 points, respectively), indicating that both geometric and progressive terms matter for contour quality.

### 4.8 Computational profile

**Table 12** reports the computational footprint for a 128 × 128 × 64 patch. HSV-Net is shown in bold. FLOPs were calculated using fvcore with OrthoLoRA stride-2 slicing and HGM on the 16 × 16 × 8 token lattice; latency follows the synchronized protocol described in the Implementation section. **Table 13** decomposes HSV-Net FLOPs. With stride-2 OrthoLoRA on a 128 × 128 × 64 crop, the stream evaluates 64*/*2 = 32 axial, 128*/*2 = 64 coronal, and 128*/*2 = 64 sagittal slices (≈ 160 DINOv2 ViT-B/14 forward), accounting for 39.8 G of the 41.0 G OrthoLoRA budget; LoRA adapters and trilinear back-projection/averaging add 1.2 G. The remaining 48.4 G are attributable to SSM3D (38.2 G) and HGM+FPN (10.2 G), for a total of 89.4 G. The reported 58 ms latency matches this single-pass graph under the Implementation timing protocol and excludes EdgeRefiner.

**Table 12:** Computational footprint for a 128 × 128 × 64 patch.

Method	Trainable (M)	Total (M)	FLOPs (G) ↓	VRAM (GB) ↓	Latency (ms) ↓
VOILA	24	24	58.2	6.8	**45**
3D U-Net	19	19	54.8	6.1	52
ViM-UNet	28	28	62.4	7.2	51
**HSV-Net**	**31**	**117**	**89.4**	**11.8**	**58**
nnU-Net (patch)	31	31	118.6	10.4	96
nnU-Net (full-volume)	31	31	124.3	12.6	148
SwinUNETR	62	62	342.1	14.5	142
SAM-Med3D	636	636	921.2	22.4	180

Lower (↓) is better for FLOPs/VRAM/latency.

**Table 13:** HSV-Net FLOPs breakdown for one 128 × 128 × 64 forward pass (fvcore; OrthoLoRA stride-2).

Module	FLOPs (G)	Share
SSM3D encoder	38.2	42.7%
OrthoLoRA: axial DINOv2 (32 slices)	8.0	8.9%
OrthoLoRA: coronal DINOv2 (64 slices)	15.9	17.8%
OrthoLoRA: sagittal DINOv2 (64 slices)	15.9	17.8%
OrthoLoRA: LoRA + back-projection	1.2	1.3%
HGM + FPN decoder	10.2	11.4%
**Total (inference graph)**	**89.4**	100%
EdgeRefiner (*T* = 3; training only)	1.8	—

Only 0.3% of DINOv2 weights are trainable via LoRA, but the frozen backbone still contributes to total size and inference memory (117 M total). Relative to SAM-Med3D, HSV-Net reduces FLOPs by 10.3× and VRAM by 47%. Versus ViM-UNet, it adds trainable capacity and 27 G FLOPs but gains 6.0 points for Dice and 8.5 points for Boundary F1 on QIN. Training fits on one RTX 3090 (11.8 GB). Inference reaches approximately 17 patches/s for a single 128 × 128 × 64 crop. OrthoLoRA still evaluates ≈ 160 2D ViT forward per crop (stride-2, three planes), so the cost scales approximately linearly with the number of extracted slices and with the number of nodules processed as separate crops. Doubling the depth from 64 to 128 slices raises measured latency to 108 ms; a 160 × 160 × 80 crop uses 17.2 GB and 91 ms (**Table 14**). Processing *K* nodules therefore requires roughly *K* independent patch forward unless a shared full-volume OrthoLoRA cache is engineered, which we leave for future systems work.

**Table 14:** Patch-size sensitivity for HSV-Net on QIN (Dice/Boundary F1% ↑).

Patch size	Dice (%)	Boundary F1 (%)	VRAM (GB)	Latency (ms)
96 × 96 × 48	88.3	83.9	8.4	41
128 × 128 × 64 (default)	**90.1**	**86.3**	11.8	58
160 × 160 × 80	90.5	86.6	17.2	91

Bold values indicate the best result in each column.

### 4.9 Robustness and input perturbations

We re-evaluated frozen QIN models on corrupted test patches (**Figure 6**, **Table 15**) using Gaussian noise (*σ* ∈ {10,25} HU), intensity shifts (± 50 HU), and anisotropic resampling to 1.0 × 1.0 × 2.5 mm before returning to the training spacing. HSV-Net retained the highest Dice in all settings. The largest drop was 3.4 points under *σ* = 25 noise (vs. 5.1 for SAM-Med3D and 6.8 for SwinUNETR); anisotropic resampling reduced Dice by 2.5 points (vs. 4.4 for SwinUNETR). Rank order was unchanged across all perturbations.

**Figure 6:**
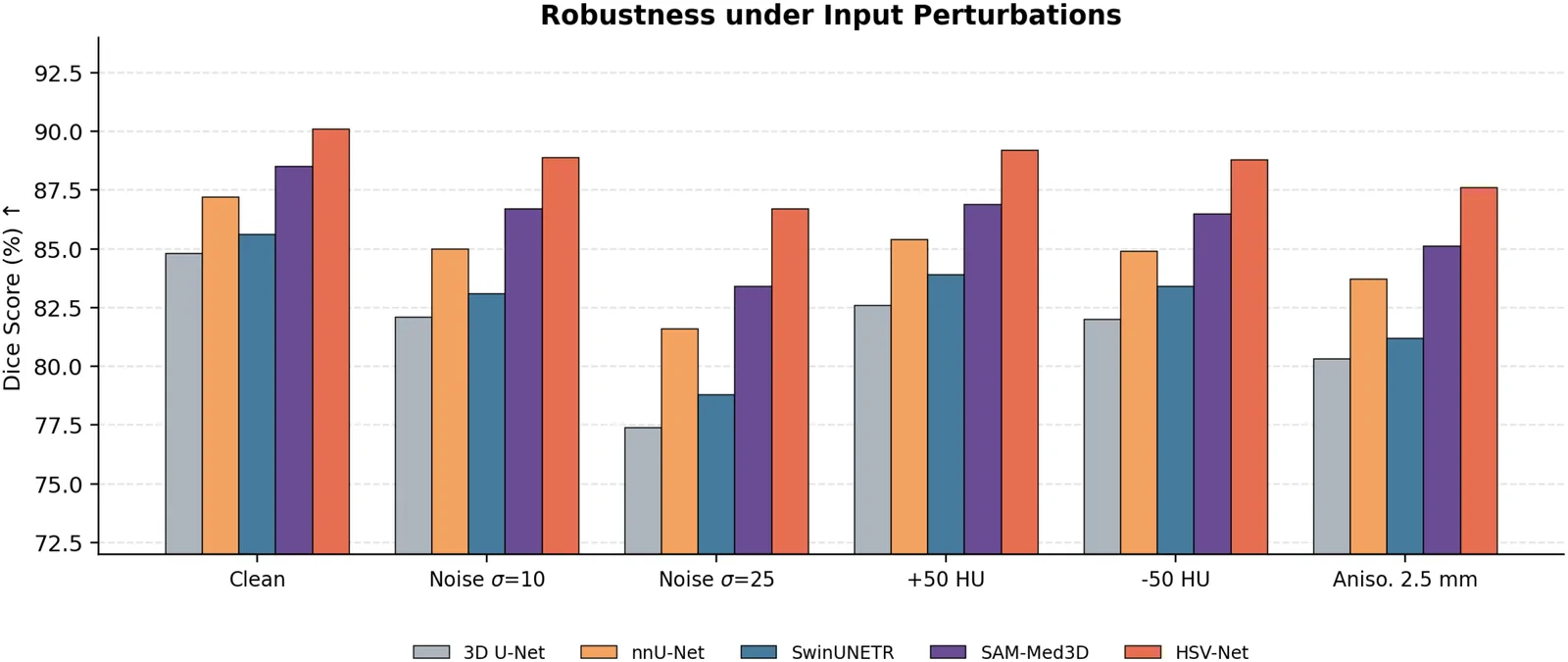
Robustness of Dice under noise, intensity shift, and anisotropic resampling (QIN-trained models, no fine-tuning).

**Table 15:** Dice (%) ↑ under input perturbations on QIN outer test folds.

Condition	3D U-Net	nnU-Net	SwinUNETR	SAM-Med3D	HSV-Net
Clean	84.8	87.2	85.6	88.5	**90.1**
Noise *σ* = 10 HU	82.1	85.0	83.1	86.7	**88.9**
Noise *σ* = 25 HU	77.4	81.6	78.8	83.4	**86.7**
Intensity +50 HU	82.6	85.4	83.9	86.9	**89.2**
Intensity −50 HU	82.0	84.9	83.4	86.5	**88.8**
Anisotropic 2.5 mm slices	80.3	83.7	81.2	85.1	**87.6**

**Table 3** reports Dice after shifting the crop center at inference (Gaussian jitter, no retraining). Mild localization error (*σ_c_* = 2 mm) reduced HSV-Net Dice by 0.8 points; *σ_c_* = 6 mm reduced it by 3.1 points, although performance remained above the clean 3D U-Net baseline. Larger localization errors still degrade overlap, confirming that upstream detection accuracy remains a prerequisite for the patch-based cascade.

### 4.10 Patch-size sensitivity

**Table 14** reports HSV-Net performance when the crop size was varied while keeping the same patient folds and training budget. The default 128 × 128 × 64 setting offered the best accuracy–memory trade-off. Smaller 96 × 96 × 48 patches reduced context for juxta-vascular cases (−1.8 points for Dice, −2.4 points for Boundary F1). Larger 160 × 160 × 80 patches slightly improved Dice (+0.4 points) but increased VRAM to 17.2 GB and latency to 91 ms, approaching the memory ceiling of a 24 GB GPU under a batch size of 2. We therefore retain 128 × 128 × 64 as the operating point for all main comparisons.

### 4.11 Independent benchmark on LIDC-IDRI

We next evaluated HSV-Net on the independent LIDC-IDRI benchmark described in Section 3.6 (186 patients and 294 nodules; expert ≥ 3*/*4 radiologist consensus masks; patient-level 5-fold CV within LIDC, with no QIN overlap and no QIN→LIDC weight transfer). **Table 2** reports mean outer-fold metrics. HSV-Net achieved the highest Dice (87.8% ± 1.1%) and Boundary F1 (83.6% ± 1.3%), outperforming full-volume nnU-Net by 0.7 and 1.4 points, respectively. Absolute scores were lower than those obtained on the small QIN development cohort, as expected for a larger, multi-scanner independent benchmark. Full-volume nnU-Net again exceeded its patch counterpart, but contour metrics still favored HSV-Net, consistent with PCL emphasizing peripheral agreement. These results support the method ranking beyond the ten-patient QIN subset while acknowledging that zero-shot QIN→LIDC transfer, multi-institutional reader studies, and longitudinal endpoints remain outside the present scope.

### 4.12 External evaluation on LUNA16

LUNA16 (47) provides centroids and diameters but not official voxel-level segmentations. Diameter-matched spherical reference masks centered at the annotated coordinates were therefore used only as a proxy size/localization transfer check, not as a valid contour benchmark: real nodules are irregular and spiculated, so Boundary F1 and HD95 against spheres are not clinically interpretable contour scores. From LUNA16 subsets 0–2, we retained nodules with diameters of 3–30 mm, complete CT availability after isotropic resampling, and a unique centroid inside the lung parenchyma, yielding 89 of 356 annotated nodules. **Table 16** reports overlap against these spheres after applying QIN-trained models without fine-tuning; we interpret the table strictly as a relative ranking under a matched proxy protocol. Expert-mask evaluation on the independent LIDC-IDRI benchmark is reported separately (**Table 2**).

**Table 16:** LUNA16 proxy transfer against diameter-matched spherical references (no fine-tuning).

Method	Dice (%) ↑	Boundary F1 (%) ↑	HD95 (mm) ↓
3D U-Net	81.4	73.8	8.6
nnU-Net (patch)	84.6	78.2	7.5
SwinUNETR	82.8	75.4	8.4
SAM-Med3D	86.4	80.1	7.2
**HSV-Net**	**88.2**	**83.9**	**6.6**

Boundary metrics are not valid contour scores.

Relative to QIN, HSV-Net Dice under this proxy protocol fell by 1.9 points. Stratified LUNA tables from the prior submission were removed for the same underpowered-subgroup reason noted above.

### 4.13 Qualitative comparison

**Figure 7** shows representative comparisons on LUNA16 patches (using the same preprocessing as for training). Green contours denote the diameter-matched spherical reference masks used for scoring.

**Figure 7:**
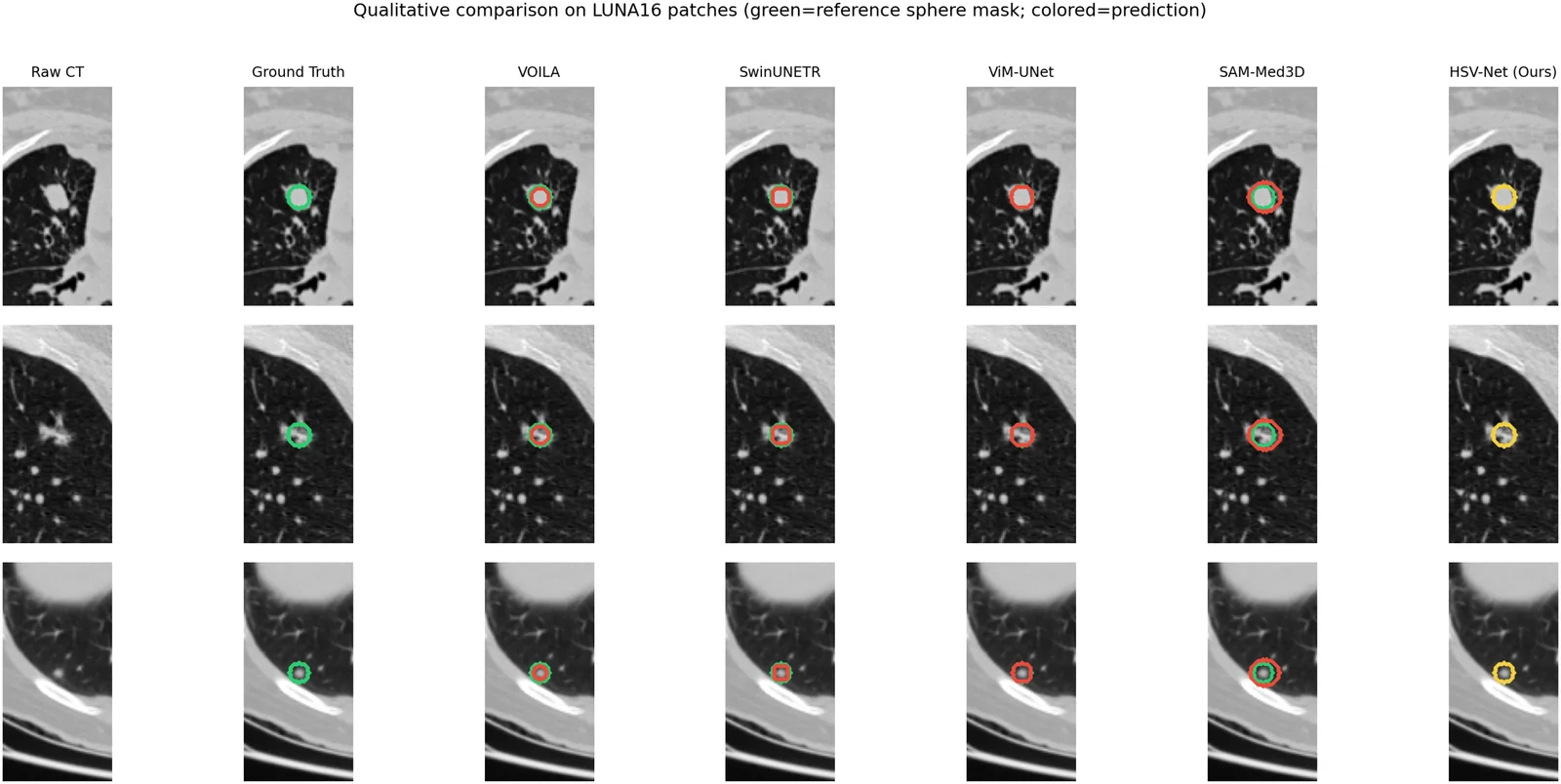
Qualitative comparison on LUNA16 patches (larger panels). Green: diameter-matched spherical reference; colored overlays: model predictions. Columns correspond to VOILA, SAM-Med3D, and HSV-Net.

**Case 1 (compact solid-appearing).** VOILA produced overly rounded contours that missed irregular margin detail, whereas HSV-Net stayed closer to the reference boundary, consistent with its higher Boundary F1 reported in **Table 4**.
**Case 2 (near vessels).** SAM-Med3D expanded into adjacent high-intensity structures, a pattern that would inflate volume estimates in serial follow-up. HSV-Net reduced this leakage relative to the reference mask.
**Case 3 (lower contrast).** Baseline methods tended to under-cover faint peripheral voxels. HSV-Net recovered a larger contiguous region in this example.

## 5 Discussion

HSV-Net addresses a practical need in thoracic cancer imaging: localized, post-detection nodule segmentation for quantitative assessment modules in CAD-style pipelines. Dual-stream fusion targets complementary failures—weak volumetric continuity and weak local contrast—while PCL improves contour metrics on the QIN-LungCT-Seg consensus labels. We evaluate HSV-Net as a compact segmentation module after nodule localization within a detection-and-segmentation cascade, aligned with localize-then-refine practices in cluttered small-object settings (13; 14; 15).

Boundary F1, HD95, and volume-derived errors (**Table 8**) suggest that gains concentrate at lesion peripheries rather than only in the nodule interior in this development cohort. On the independent LIDC-IDRI benchmark (**Table 2**), HSV-Net retained the best contour metrics despite lower absolute Dice scores than on QIN, supporting generalization beyond the ten-patient subset. The full-volume nnU-Net baseline (**Table 6**) closed part of the gap relative to patch-adapted nnU-Net but did not change the ranking on either cohort. Perturbation and centroid-jitter tests (**Tables 15**, **3**) probe stability under noise, anisotropic resampling, and imperfect localization; degradation-aware and intensity-normalized dual-branch designs similarly emphasize robustness when appearance is non-uniform (14; 37). The 58 ms latency and 11.8 GB footprint are compatible with workstation CAD alongside detection for single localized crops, subject to the OrthoLoRA scaling caveats described above.

Compared with SAM-Med3D under matched patch crops, HSV-Net matched or exceeded overlap and boundary scores on QIN at lower latency, while SAM-Med3D additionally required ground-truth boxes at inference. Ablations (**Tables 9**, **10**) show that boundary loss alone plateaued below PCL; placing short multistep refinement during training improves uncertain margin voxels without requiring multipass inference. Efficient selective-state detectors reinforce that Mamba-style scanning can capture fine structures under constrained computational resources (21). Cross-stream fusion between mismatched representations likewise benefits from designs that transfer and align multi-view features rather than relying on naive stacking (39; 37). Privacy-preserving multi-institutional oncology modeling remains complementary to single-center CAD modules when data cannot leave hospital boundaries (32; 31).

Limitations. The primary QIN cohort remains small (10 patients and 12 tumors) and is intended for nested method development rather than powered subgroup analysis; morphology- and size-stratified analyses were therefore omitted. LUNA16 results use spherical proxies and must not be interpreted as contour external validation. The LIDC-IDRI study is an independent within-dataset benchmark rather than a zero-shot transfer of QIN-trained models, and still lacks the multi-institutional reader agreement and longitudinal outcomes needed to support deployment claims. Loss weights and LoRA/*T* choices were fixed from a two-patient inner split and may be overfit. Longitudinal growth, Lung-RADS outcomes, and reader studies were not evaluated. Future work will prioritize QIN→LIDC transfer without retraining, multisite reader evaluation, and end-to-end detector-driven localization.

## 6 Conclusions

We presented HSV-Net, which combines volumetric state-space encoding, OrthoLoRA adaptation of DINOv2, and progressive contour learning for CT pulmonary nodule segmentation after localization. On the small QIN development subset with nested patient-level evaluation, it achieved the best overlap and boundary scores among the compared methods, including a standard full-volume nnU-Net v2 baseline. On an independent LIDC-IDRI benchmark with expert volumetric consensus masks, HSV-Net likewise led in Dice and Boundary F1 while maintaining favorable latency relative to larger foundation-model baselines. Clinical translation will still require multi-institutional reader studies and longitudinal validation.

## Data Availability

The QIN Lung CT images, QIN-LungCT-Seg masks, LIDC-IDRI, and LUNA16 annotations are publicly available via TCIA and the LUNA16 challenge repository. Patient identifiers for the ten-case Moffitt subset, fold assignments, preprocessing scripts, training configurations, inference code, and trained weights for all five outer folds are available from the corresponding author.
